# AmyP53, a Therapeutic Peptide Candidate for the Treatment of Alzheimer’s and Parkinson’s Disease: Safety, Stability, Pharmacokinetics Parameters and Nose-to Brain Delivery

**DOI:** 10.3390/ijms232113383

**Published:** 2022-11-02

**Authors:** Coralie Di Scala, Nicholas Armstrong, Henri Chahinian, Eric Chabrière, Jacques Fantini, Nouara Yahi

**Affiliations:** 1 Neuroscience Center—HiLIFE, Helsinki Institute of Life Science, University of Helsinki, 00014 Helsinki, Finland; 2 IRD, APHM, MEPHI, IHU Méditerranée Infection, Aix Marseille University, 13005 Marseille, France; 3 INSERM UMR_S 1072, Aix Marseille University, 13015 Marseille, France

**Keywords:** Alzheimer’s, Parkinson’s, therapy, peptide, ganglioside, amyloid pore

## Abstract

Neurodegenerative disorders are a major public health issue. Despite decades of research efforts, we are still seeking an efficient cure for these pathologies. The initial paradigm of large aggregates of amyloid proteins (amyloid plaques, Lewis bodies) as the root cause of Alzheimer’s and Parkinson’s diseases has been mostly dismissed. Instead, membrane-bound oligomers forming Ca^2+^-permeable amyloid pores are now considered appropriate targets for these diseases. Over the last 20 years, our group deciphered the molecular mechanisms of amyloid pore formation, which appeared to involve a common pathway for all amyloid proteins, including Aβ (Alzheimer) and α-synuclein (Parkinson). We then designed a short peptide (AmyP53), which prevents amyloid pore formation by targeting gangliosides, the plasma membrane receptors of amyloid proteins. Herein, we show that aqueous solutions of AmyP53 are remarkably stable upon storage at temperatures up to 45 °C for several months. AmyP53 appeared to be more stable in whole blood than in plasma. Pharmacokinetics studies in rats demonstrated that the peptide can rapidly and safely reach the brain after intranasal administration. The data suggest both the direct transport of AmyP53 via the olfactory bulb (and/or the trigeminal nerve) and an indirect transport via the circulation and the blood–brain barrier. In vitro experiments confirmed that AmyP53 is as active as cargo peptides in crossing the blood–brain barrier, consistent with its amino acid sequence specificities and physicochemical properties. Overall, these data open a route for the use of a nasal spray formulation of AmyP53 for the prevention and/or treatment of Alzheimer’s and Parkinson’s diseases in future clinical trials in humans.

## 1. Introduction

Neurodegenerative diseases will undoubtedly be a major pandemic in this century. Alzheimer’s disease and associated dementias already affect tens of millions of people worldwide, and that number could reach more than 100 million by 2050 [1], i.e., for the next generation. For its part, Parkinson’s disease is currently affecting more than 6 million individuals in the world [2], and its occurrence has more than doubled between 1990 and 2016 [3]. These estimations underline the major impact of these pathologies for our health systems.

To date, there is no cure for these diseases. In the best of cases, physicians try to treat the symptoms but not the root causes of these diseases, despite 50 years of intense research efforts. Indeed, though the proteins responsible for these diseases have been formally identified, the precise molecular and cellular mechanisms causing these pathologies are still debated. The initial paradigm of amyloid plaques that have long been held to be responsible for Alzheimer’s disease is now abandoned. In fact, the correlation between the presence of amyloid plaques and the symptoms of Alzheimer’s disease could not be established, and many arguments have been made on the contrary to dismiss this theory. The famous “nun study”, conducted by D. Snowdon in the 1990s [4], is a particularly significant example, but it is far from being the only one. The model of amyloid plaques had been questioned as early as 1968 by Tomlison et al. [5]. Subsequently, many results reinforced the skepticism regarding amyloid plaques: the failure of anti-plaque immunotherapies [6,7,8,9,10,11], the existence of genetic forms of Alzheimer’s disease (Osaka mutation) without evidence of plaques in the brains of patients [12,13], the presence of amyloid plaques in nondemented subjects [14,15,16], and finally the discovery of amyloid protein (Aβ) oligomers and their correlation with symptoms [10,17,18,19,20,21,22,23]. A similar scenario is emerging for Parkinson’s disease, and numerous data suggest that, instead of Lewy bodies, α-synuclein oligomers are the real cause of the disease. Gaig et al. have identified a single mutation in the leucine-rich repeat kinase 2 gene (LRRK2) that causes Parkinson’s disease without Lewy bodies [24]. Several studies failed to establish a clear-cut correlation between Lewy bodies and Parkinson’s symptoms [25,26], whereas a large body of evidence suggest that α-synuclein oligomers are the real culprits [27,28,29,30,31,32,33].

**Figure 1:**
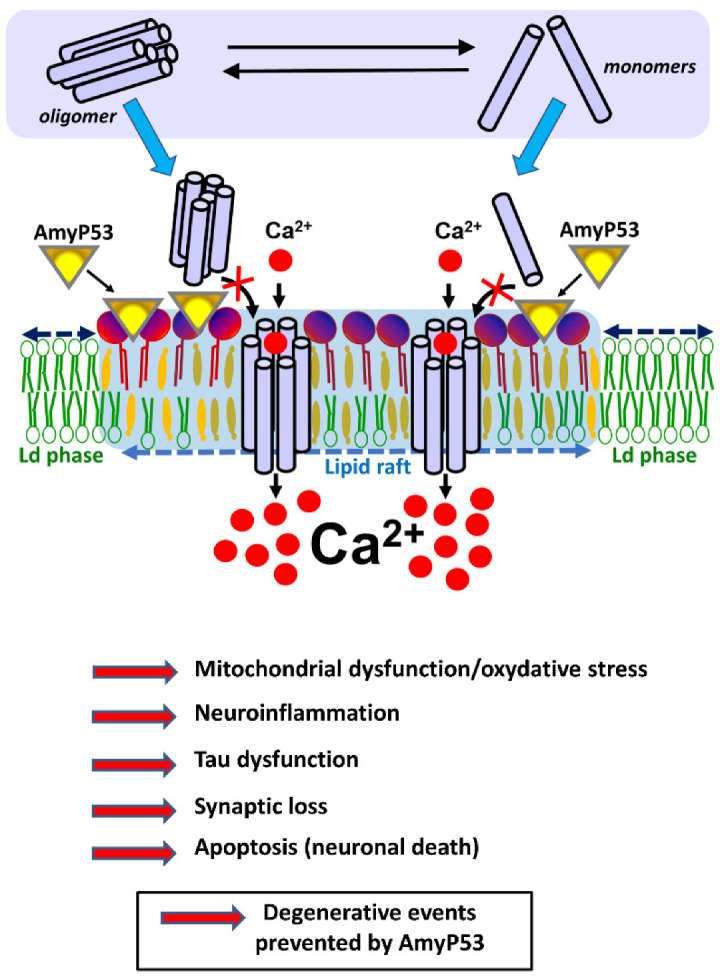
AmyP53 blocks a mechanism of neurotoxicity shared by both Alzheimer’s and Parkinson’s diseases. AmyP53 blocks the neurotoxic cascade triggered by oligomers in the membrane of brain cells. Amyloid pores may be either preassembled in the extracellular space (soluble oligomers, in equilibrium with monomers and intermediate assemblies) or within the plasma membrane of brain cells (from monomers that bind to the membrane). Yet in both cases the formation of these amyloid pores requires gangliosides that act as specific membrane receptors. Ca^2+^ ions rush into these pores, triggering a cascade of neurotoxic events that disrupt brain activity and precipitate the disease in patients. By preventing any amyloid protein from binding to gangliosides, AmyP53 blocks the overall neurotoxicity cascade at this earliest membrane step that is common to both Alzheimer’s and Parkinson’s diseases.

Both Aβ and α-synuclein oligomers result from the self-assembly of a few molecules of amyloid proteins according to a perfectly determined and structured mode of building, whereas the formation of amyloid plaques is rather stochastic. Many types of oligomers have now been described, but they can be schematically classified into two main groups: soluble oligomers [34,35] and membrane-associated oligomers [36,37,38]. Soluble oligomers that do not interact with brain cells do not appear to be toxic, at least as long as they remain soluble in the extracellular milieu. On the other hand, they can become highly neurotoxic when they interact with the plasma membrane of a neuron or a glial cell [39,40]. As a matter of fact, the toxicity of oligomers is typically a membrane phenomenon, which suggests that Alzheimer’s disease is primarily a membrane disorder [41,42]. The interaction of these oligomers with the membrane of nerve cells is mediated via special glycolipids called gangliosides, which are particularly abundant in the central nervous system [43,44]. Gangliosides are grouped together in cholesterol-enriched microdomains of the membrane, thus forming particular structures referred to as lipid rafts [45,46]. During the last years, lipid raft gangliosides have thus been identified as a prime target for developing new therapeutic strategies for neurodegenerative diseases [47,48,49,50,51]. The selection of gangliosides as attachment sites is shared by many amyloid proteins, including Alzheimer’s disease Aβ peptide (Aβ) [52,53,54] and Parkinson’s disease α-synuclein [55,56,57,58]. Whether in the form of preformed oligomers, or monomers that first insert into the membrane and then self-assemble into oligomeric structures, these proteins must first bind to a ganglioside [39]. Membrane-inserted oligomers are referred to as “amyloid pores” [59,60]. Amyloid pores are small channels that let Ca^2+^ ions enter the cell in a permanent and nonregulated process, which is the primary cause of neurotoxicity [60]. Indeed, Ca^2+^ ions trigger a downstream cascade of events including tau hyperphosphorylation [61,62,63], oxidative stress [64,65], synaptic deterioration [66,67,68,69], plasticity dysfunction [70], and eventually cell death [29,71,72]. Thus, targeting gangliosides and occupying their surface to prevent amyloid proteins from attaching to them and form amyloid pores is a particularly interesting and innovative therapeutic strategy [60,73]. For this purpose, it is necessary to have therapeutic molecules recognizing the gangliosides and devoid of toxicity by themselves. The AmyP53 peptide has been specifically designed for this purpose [74]. Based on 20 years of research in molecular neurosciences having led to the elucidation of the mechanisms of neurotoxicity of Aβ and α-synuclein, this peptide combines in its molecular structure the ganglioside binding properties of these two proteins, i.e., schematically GM1 for Aβ and GM3 for α-syn [74] (Figure 1).

To achieve this goal, we first identified the domains responsible for the recognition of gangliosides in the structure of Aβ and α-syn proteins. By testing a series of recombinant proteins and synthetic peptides derived from distinct parts of these proteins and by measuring their ability to bind to gangliosides, we were able to identify amino acids Aβ_5-16_ and α-syn_34-45_ as functional ganglioside-binding domains (GBDs) [57,74]. We showed that Aβ_5-16_ preferentially binds to ganglioside GM1, while α-syn_34-45_ rather recognizes GM3, just like the whole parental proteins do. We then used in silico modeling approaches to design a chimeric peptide combining the ganglioside-binding properties of both Aβ and α-syn [74]. Apart from the critical parameters controlling the recognition of gangliosides, an important criterion was water solubility, with the idea of developing a nasal-spray formulation. We also took into account the surface electrostatic potential of the chimeric peptide, which was deliberately electropositive [74] to be attracted by the electronegative field of ganglioside-enriched lipid rafts [45]. Thus, the AmyP53 peptide was designed as a competitive inhibitor of the binding of amyloid proteins to their ganglioside receptors, with a kinetic advantage over these proteins. It was also conceived as a flexible peptide lacking a secondary structure in water, thus mimicking the conformational freedom of the amyloid proteins from which it was derived. We have avoided introducing any potential proteolytic cleavage site that would decrease the in vivo stability and bioavailability of AmyP53. Taking into account these strict specifications, we constructed a chimeric peptide on the backbone of the GBD of α-syn, and we substituted amino acids 9 and 10 with a pair of histidines conferring the specificity of interaction of Aβ for GM1 [74]. It is important to note that the GBDs of Aβ and α-syn are located outside the neurotoxic domains of these proteins, so that the chimeric peptide lacks the capacity of amyloid pore formation [39,40].

By targeting a molecular mechanism common to both Alzheimer’s disease and Parkinson’s disease, AmyP53 is thus the first representative of a new class of molecules with high therapeutic potential [39,40]. We have previously shown the effectiveness of AmyP53 at blocking the binding of amyloid proteins in different experimental models (in vitro and ex vivo), whether in the form of a chemically synthesized peptide [60,73] or delivered biologically by a viral vector [75]. In particular, the therapeutic efficacy of AmyP53 was tested in an ex vivo model of Alzheimer’s disease based on Aβ-associated synaptic perturbations of brain hippocampal slices that can be assessed in real-time by electrophysiological recordings. Remarkably, the addition of equimolar concentrations of AmyP53 in competition with Aβ proteins was able to prevent the electrophysiological perturbations of brain hippocampal slices [73]. Toxicology studies in rats have then demonstrated the perfect tolerance of AmyP53 administered intravenously or intranasally [75]. In the present study, we provided the first preclinical results of the pharmacokinetics of the AmyP53 peptide in rats. We show that AmyP53 is remarkably stable in aqueous formulation, that it effectively crosses the blood–brain barrier, and that it can be delivered to the brain after either intravenous or intranasal administration. Taken together, these new results are an important step in the development of AmyP53 as a nasal-spray formulation for the treatment of Alzheimer’s and Parkinson’s diseases.

## 2. Results

### 2.1. Chemical Stability of AmyP53 in Water Solution

**Figure 2:**
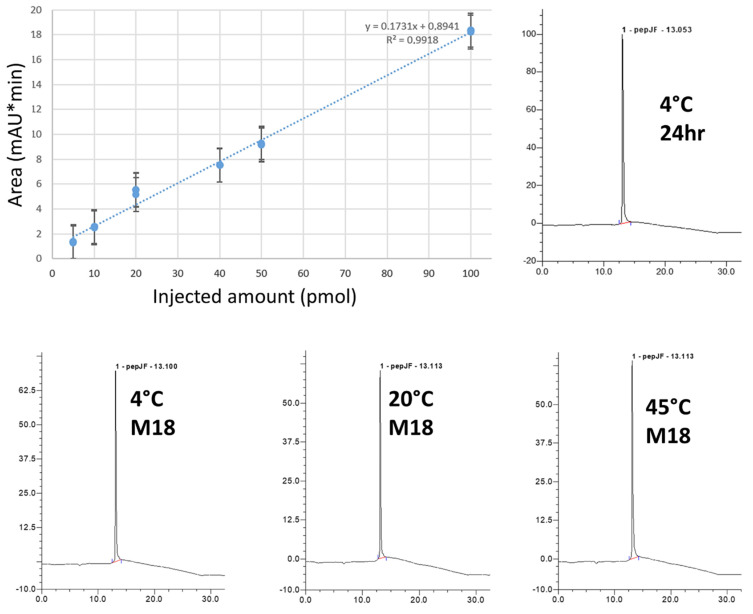
Chemical stability of AmyP53 in aqueous solution. AmyP53 was dissolved in pure water at a concentration of 1 mM and stored for 18 months at 4 °C, 20 °C, and 45 °C. The calibration curve (upper left) is linear over the 5–100 pmol range (R^2^ > 0.99). The minimal amount detected is 5 pmol. Representative original raw HPLC spectra at 24 h (upper right) and M18 (18-month, lower panels) are shown.

The main physicochemical properties of the AmyP53 peptide are listed in Table 1. The stability of AmyP53 solutions prepared in pure water was analyzed by LC–MS. A calibration curve demonstrated that this method allowed the precise quantitative determination of the amount of AmyP53 over the 5–100 pmol range (Figure 2). The chemical stability of AmyP53 solutions was analyzed at 4 °C, 20 °C, and 45 °C over a total period of 18 months. AmyP53 was analyzed at several time points during this incubation (24 h, 48 h, 72 h, 16 days, two months, and finally 18 months). We did not detect any evidence of peptide degradation at any time, and the HPLC profiles after 18 months of incubation at 4°C, 20 °C, and 45 °C were remarkably similar to the initial spectra (Figure 2). The quantification of AmyP53 did not show any variation from the reference. Thus, these data indicate that AmyP53 remains chemically intact over 18 months in water solution.

### 2.2. Biological Activity of AmyP53 Solutions upon Long-Term Storage

**Figure 3:**
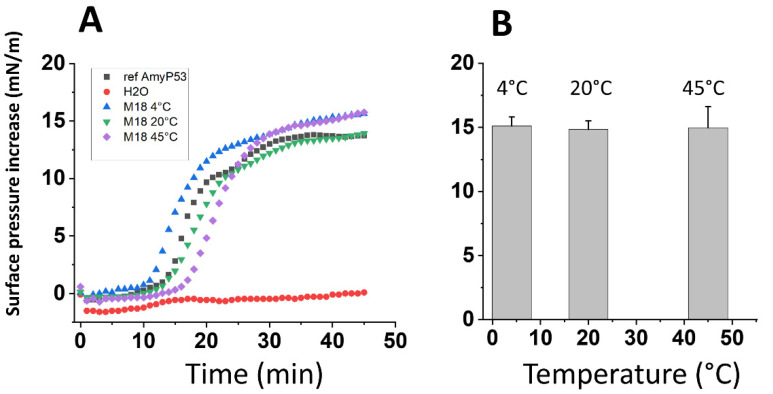
Biological stability of AmyP53 in aqueous solution. AmyP53 was dissolved in pure water at a concentration of 1 mM and stored for a maximal time of 18 months at 4 °C, 20 °C, and 45 °C. (**A**) After 18 months (M18) of storage at the indicated temperature, samples were assayed in the GM1 binding assay. Pure water was used as negative control for the Langmuir monolayer assay. (**B**) Comparison of AmyP53 binding to GM1 over a 18-month period at 4 °C, 20 °C, and 45 °C. Samples were analyzed at 24 h, 48 h, 72 h, 16 days, 2 months, and 18 months. The surface pressure increase induced by 8 µL of each sample added underneath a stable monolayer of ganglioside GM1 was measured at the end of the experiment. Data are expressed as mean ± SD (n = 6). The differences between AmyP53 concentrations at 4 °C, 20 °C, and 45 °C were statistically not significant (*p* > 0.05, Kruskal–Wallis test).

This chemical analysis was completed by a physicochemical assay to evaluate the biological activity of AmyP53 solutions over the 18-month storage at 4 °C, 20 °C, and 45 °C. The assay is based on the ganglioside-binding properties of AmyP53. When added underneath a monolayer of ganglioside GM1, the AmyP53 peptide induces a progressive increase of the surface pressure, which is the proof of a molecular interaction between the peptide and the ganglioside. This method is highly specific and sensitive, so that any decrease in the ganglioside-binding properties of AmyP53 can be demonstrated by this assay. The data in Figure 3A show the kinetics of interaction of various samples of AmyP53, including a calibrated reference solution and the solutions stored for 18 months at 4 °C, 20 °C, and 45 °C. Both the typical sigmoidal shape of the curve and the maximal surface pressure increase appeared to be similar between the reference and the tested solutions. Moreover, the compilation of all determinations at the three temperatures over the 18-month incubation time demonstrated a similar ganglioside-binding activity for all samples, whatever the storage temperature (Figure 3B). Overall, these data indicate that long term storage at 4 °C, 20 °C, or 45 °C did not impair the ganglioside-binding properties of AmyP53 solutions that remained remarkably constant during the experiment.

### 2.3. AmyP53 Stability in Blood and Serum

**Figure 4:**
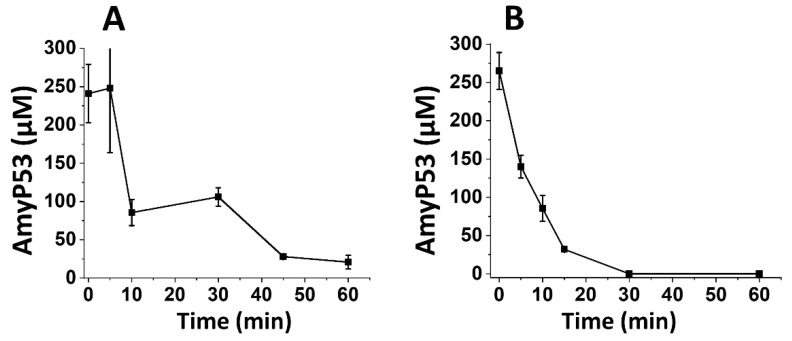
In vitro stability studies of AmyP53 in rat whole blood and human serum. AmyP53 was incubated at a concentration of 300 µM with rat whole blood (**A**) or human serum (**B**). At the indicated times, samples were extracted and underwent AmyP53 quantification by LC–MS. Data are expressed as mean ± SD (n = 3).

Then we studied the stability of AmyP53 in whole blood and serum. To this end, we initially tested two extraction protocols (methanol and trichloroacetic acid) to determine the best approach for dosing AmyP53. Methanol extraction was selected because it appeared to be more sensitive than trichloroacetic acid and trichloroacetic acid was less suitable for LC/MS analyses. For whole blood extraction, a sonication step was included in the procedure as it increases the yield of peptide recovery. Under these conditions, we could reproducibly detect and quantify AmyP53 in rat fresh-blood samples by LC–MS (Figure 4A). The apparent concentration of AmyP53 started to decline after 5 min of incubation, with a half-life time of 17 min. The peptide was still detected after 60 min of incubation. In commercial human serum, the decline of apparent AmyP53 concentration occurred significantly more quickly than in blood, and the half-life time was 5 min (Figure 4B). The peptide was no longer detected in serum after 30 min of incubation.

### 2.4. Pharmacokinetics of AmyP53 in Brain and Blood

**Figure 5:**
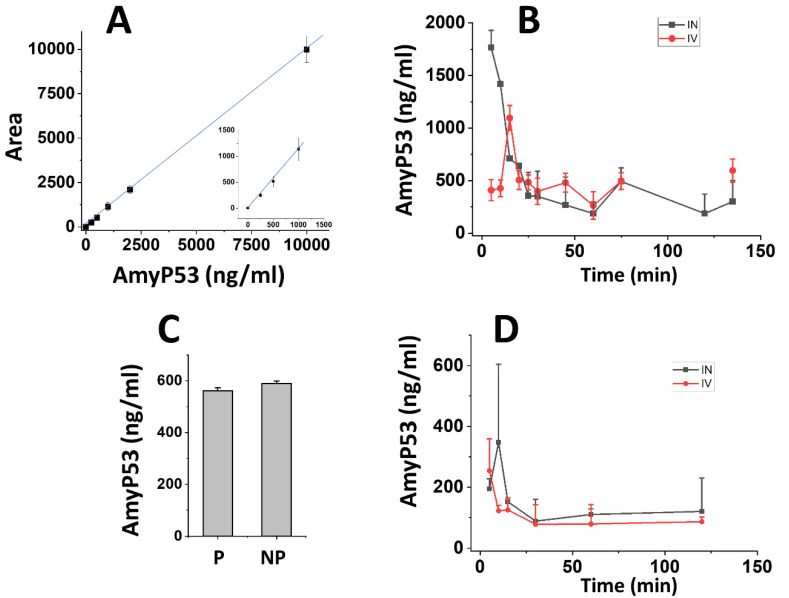
Pharmacokinetics studies of AmyP53 administered in rats by either the intravenous or intranasal routes. (**A**) Calibration curve of AmyP53 detection in brain homogenates after methanol extraction and quantification by LC–MS. The insert shows the 250–1000 ng/mL range. Data are expressed as mean ± SD (n = 4). (**B**) At the indicated time following intravenous (red symbols) or intranasal administration (black symbols), brain homogenates were prepared and extracted with the methanol method, and AmyP53 was quantified by LC–MS. Data are expressed as mean ± SD (n = 3). (**C**) Comparison of AmyP53 determinations in perfused (P) and nonperfused (NP) brain 30 min after intranasal injection. The data considered the time required for perfusion (15 min). Data are expressed as mean ± SD (n = 3). (**D**) AmyP53 quantified in whole blood samples after intravenous (red symbols) or intranasal administration (black symbols). Data are expressed as mean ± SD (n = 3). In panel C, the differences were not statistically significant (*p* > 0.05, Kruskal–Wallis test).

The pharmacokinetics parameters of AmyP53 were analyzed in rat brain and blood after either intranasal or intravenous administration (Figure 5). The method of extraction was adapted to detect the peptide in brain homogenates by the LC–MS method (Figure 5A). Intact AmyP53 was detected 5 min after intranasal administration, after which the peptide concentration regularly declined to the lowest detected values after 60 min (Figure 5B). However, the peptide was still detected after two hours. In comparison, the time required for AmyP53 to reach its maximal concentration (Cmax) in the brain after intravenous administration was 15 min, i.e., 3 times longer than via the intranasal pathway (5 min) (Figure 5B). The pharmacokinetics parameters of the two administration pathways are listed in Table 2. In summary, the intranasal route had a higher Cmax (1766 ng/mL) and shorter Tmax (5 min) compared with the intravenous pathway (Cmax = 1095 ng/mL, Tmax = 15 min). Based on the AUC determinations of the pharmacokinetics study (Table 2) and considering the amounts of AmyP53 injected, we calculated that 1.57% of AmyP53 reached the brain after intranasal administration and 0.2% after intravenous injection. Thus, nose-to brain delivery was 7.85-fold more efficient than blood circulation for AmyP53.

To ensure that the AmyP53 detected in the brain was recovered from brain tissue and not from brain vessels, a control experiment was performed that included brain perfusion before homogenate preparation. Since the perfusion protocol required 15 min, it was not possible to study the pharmacokinetics of AmyP53 during the first time points. However, we could compare the amount of Amy53 detected in the perfused brain and compared the values obtained from the nonperfused brain at 30 min (Figure 5C). At this time, similar AmyP53 amounts were measured in both conditions, showing that the therapeutic peptide is indeed able to reach brain tissue after intranasal administration.

Finally, we studied the pharmacokinetics of AmyP53 in blood (Figure 5D). In this case, the peptide peaked immediately after intravenous injection, whereas there was a lag following intranasal administration. In both cases, the amount of AmyP53 recovered in blood was lower than the amount detected in brain. A major outcome of these pharmacokinetics studies is that AmyP53 is able to reach the brain after either an intranasal or an intravenous treatment. In the latter case, the data strongly suggest that the peptide is able to cross the blood–brain barrier. Since transport through the blood–brain barrier is of high interest for the development of a therapeutic drug, we decided to conduct a series of experiments to document the blood–brain-barrier-penetration capability of AmyP53.

### 2.5. Kinetics of AmyP53 Transport through an In Vitro Model of the Blood–Brain Barrier

**Figure 6:**
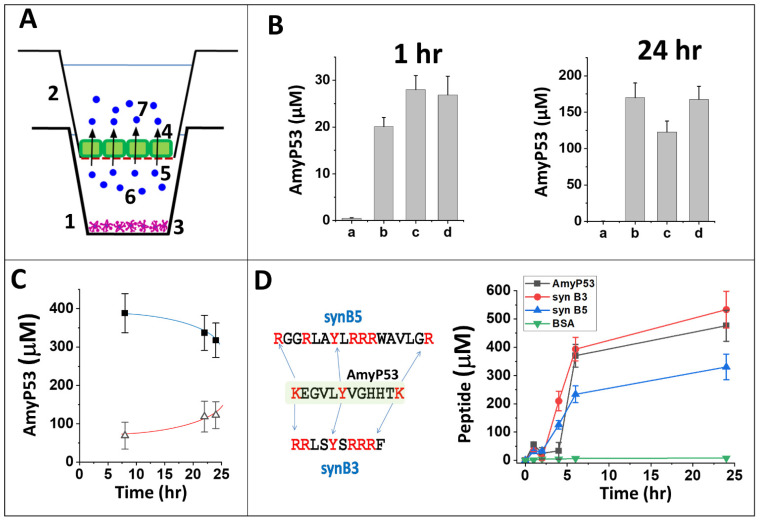
Passage of the AmyP53 peptide through the blood–brain barrier. (**A**) Two-compartment cell culture device. At time t0, AmyP53 is injected in the basal (donor) compartment (1), and it is progressively transported into the apical (acceptor) compartment (2). When indicated, CTX or C6 cells (3) are seeded in the basal compartment (non-contact model). The endothelial bEnd.3 cells (represented in green) (4) colonize the permeable filter (red dashed line) (5) that separates both compartments. AmyP53 molecules in the donor and acceptor compartments are respectively noted (6) and (7). (**B**) Detection of AmyP53 in the acceptor compartment after 1 h and 24 h of incubation. The cell models are bEnd.3 cells (a, b), bEnd.3/CTX (c), or bEnd.3/C6 (d). PBS (a) or AmyP53 (b, c, d) was added in the basal compartment. Data are expressed as mean ± SD (n = 6). (**C**) Simultaneous detection of AmyP53 in the donor (full squares, blue curve) and acceptor (open triangles, red curve) compartments. The data show the typical simultaneous disappearance of AmyP53 from the basal donor compartment and its progressive appearance in the apical acceptor compartment (bEnd.3/C6 model). (**D**) Comparison of AmyP53 transendothelial transport (black symbols) with two cargo peptides (synB3, red symbols, and synB5, blue symbols) and bovine serum albumin (BSA, green symbols) (bEnd.3/C6 model) in a typical experiment. The amino acid sequence of AmyP53 vs. syn B3 and syn B5 is shown in the left panel. Data are expressed as mean ± SD (n = 6).

The transport of intact AmyP53 through the blood–brain barrier was studied with three reconstituted in vitro cellular models cultured in a two-compartment chamber [76] (Figure 6A). These three models consist of (i) pure bEnd.3 cells (mouse endothelial brain cells), (ii) bEnd.3/CTX (rat brain astrocytes) co-cultures, and (iii) bEnd.3/C6 (rat brain glial cells) co-cultures [77]. The impermeability of each of these cellular barriers was assessed by transendothelial resistance measurements. Experimental data have shown that an electrical resistance > 100 Ω.cm^2^ is sufficient to prevent the paracellular passage of ions [78], radioactive iodine [79], and sodium fluorescein (a compound with a Mw of 376 Da, i.e., 3–4 fold smaller than the AmyP53 peptide) [80], reflecting the presence of functional tight junctions on the entire surface monolayer [78,81,82]. The transendothelial resistance measurement results for the three models are as follows: pure bEnd.3 cells, 185 ± 24 Ω.cm^2^; bend-3/CTX system, 153 ± 18 Ω.cm^2^; bend-3/C6 system, 186 ± 22 Ω.cm^2^. Although higher values (>1000 Ω.cm^2^) have been recorded with induced pluripotent stem-cell-derived endothelial cells (iPSC-ECs) [83], which may be more representative of the in vivo conditions, these three reconstituted systems fulfill the minimum electrophysiological requirements for in vitro models of the blood–brain barrier [80,84]. In order to compare the transport efficiency of the AmyP53 peptide through such blood–brain barrier models, we presented the results in the form of histograms summarizing the concentration of the peptide in the acceptor compartment after 1 h and 24 h of incubation (Figure 6B). The comparison of these results shows a remarkable reproducibility in the three systems studied. bEnd.3/C6 co-cultures gave the best combined results at 1 h and 24 h. The experiment in the presence of PBS alone (without peptide) shows that bEnd.3 cells do not secrete any factor that interferes with our peptide assay protocol of dosing. Moreover, we carefully checked that the transendothelial resistance values remained unchanged during the incubation with AmyP53, which demonstrates that AmyP53 did not impair the leakproof properties of this reconstituted blood–brain barrier.

Based on these data, we selected bEnd.3/C6 co-cultures as our reference model for studying the kinetics of AmyP53 transport through the blood–brain barrier. Firstly, we observed that the passage of the peptide resulted in a decrease in the concentration in the donor compartment associated with a progressive increase in the acceptor compartment (Figure 6C). Secondly, we demonstrated that bovine serum albumin (BSA), a non-permeant high-molecular-weight protein, did not cross the blood–brain barrier at all, illustrating the high selectivity of AmyP53 transport (Figure 6D). Thirdly, we analyzed two short polybasic peptides (cell penetrating peptides) used as cargo vehicles to transport therapeutic molecules from the blood to the brain via the blood–brain barrier (Figure 6D). These two peptides, synB3 and synB5 [85], display a central tyrosine residue and several lateral (or terminal) basic amino acids, i.e., sequence characteristics that are also shared with AmyP53. Both synB3 and synB5 efficiently crossed the blood–brain barrier but with markedly different kinetics, which argues against a paracellular free diffusion. The AmyP53 peptide appeared to be almost as effective as synB3 and more than synB5. These data fit with the analysis via the C2Pred server, a sequence-based tool for identifying cell-penetrating peptides (CPP) [86], which predicted a CPP probability >90% for AmyP53. 

### 2.6. Biological Activity of AmyP53 after Blood–Brain-Barrier Transport

**Figure 7:**
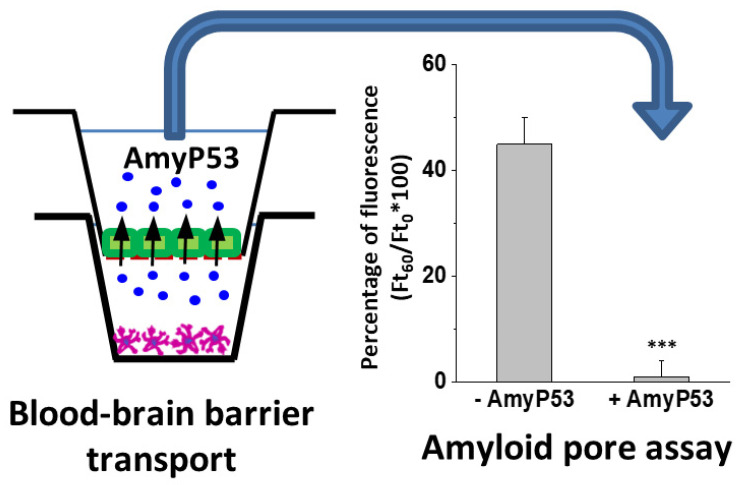
Therapeutic efficiency of AmyP53 recovered after blood–brain-barrier transport (bEnd.3/C6 model). AmyP53 was injected in the donor compartment and recovered from the acceptor compartment after 24 h of incubation (**left panel**). Samples were tested in the amyloid pore assay (**right panel**) in presence of Aβ1-42. Control supernatants (−AmyP53) did not inhibit the Ca^2+^ flux triggered by Aβ1-42, whereas AmyP53 transported through the reconstituted blood–brain barrier (+AmyP53) was fully active. Data are expressed as mean ± SD (n = 6); *** indicates *p* < 0.005 (Kruskal–Wallis test).

Finally, we checked the biological activity of the AmyP53 peptide harvested in the apical compartment of the culture chambers after transport through the reconstituted blood–brain barrier (bEnd.3/C6 model). Incubated in competition with the β-amyloid 1-42 peptide, which forms amyloid pores and induces an influx of calcium ions into the SH-SY5Y cells, the transported AmyP53 peptide remained fully active: it blocked the formation of amyloid pores (Figure 7). In this experiment, calcium fluxes were measured with the Fluo-4AM probe, a fluorescent indicator sensitive to calcium concentration. Fluorescence microscopy analysis then makes it possible to follow in real time the evolution of the calcium concentrations of individual cells, to determine the calcium concentration of each of these cells and to sum the values resulting from the analysis of hundreds of cells (150 for the histograms shown in Figure 7).

Overall, these data showed that intact and biologically active AmyP53 can be transported through the blood–brain barrier, consistent with pharmacokinetics data obtained in rodents. Finally, toxicology data demonstrated the safety of AmyP53 intranasal administration over a 7-day period, confirming and extending the data of a previous study [75] (Table 3). For both male and female Wistar rats, no mortality, no behavioral changes, no body weight loss, and no alterations of brain tissue at autopsy were observed after repeated intranasal injections of AmyP53 at doses up to 5 mg/kg body weight.

## 3. Discussion

A cure for Alzheimer’s and Parkinson’s disease is urgently needed, as these neurological disorders are expected to affect more and more people over the next few years. In this study, we present the first pharmacokinetics data of AmyP53, a therapeutic peptide specifically designed to block a common root cause of both diseases, i.e., the initial binding of amyloid proteins Aβ and α-synuclein to cell membrane gangliosides [60,73]. 

The peptide nature of AmyP53 gives to this potential solution a clearcut advantage over alternative treatments such as immunotherapies, since peptides represent a unique class of pharmaceutical compounds with distinct biochemical and therapeutic characteristic [87,88]. More than 400 peptide drugs are under clinical evaluation, and 60 have already been approved for clinical use worldwide, including the US, Europe, and Japan. Among key advantages of therapeutic peptides, one can cite high specificity, lack of accumulation in tissues, and low toxicity because peptides are metabolized into non-toxic metabolites (natural amino acid components) [89,90]. Hence, there are no safety concerns after catabolism for peptides composed of only naturally occurring amino acids [91]. Consistently, peptides can reach biomolecular targets that are difficult to tackle with small molecules, which generally suffer from low affinity, poor selectivity, and low solubility in water [88]. Moreover, peptides are also superior to large proteins such as antibodies that have bioavailability issues, especially for brain diseases [92], and can also induce serious immune responses [93].

For these reasons, peptides are considered alternative therapeutic approaches for various pathologies, including neurological disorders [94]: PACAP, a neuroprotective peptide [95], p8, an inhibitor of the protease that cleaves the APP precursor and generates Aβ amyloid [96], and liraglutide, a peptide analog of glucagon-like peptide 1 [97]. Liraglutide has been shown to rescue Aβ1-42-induced Tau hyperphosphorylation and to improve memory in an animal model of Alzheimer’s disease [98]. This peptide also showed activity in mouse models of Parkinson’s disease [99,100], which illustrates the existence of common mechanisms of neuropathology shared by both Alzheimer’s and Parkinson’s diseases [60]. 

The AmyP53 peptide tackles one of these common mechanisms directly at the brain cell plasma membrane level, where both diseases may start. AmyP53 has been designed on the basis of the deciphering of the biological code controlling the interaction of amyloid proteins with gangliosides [74]. In this respect, it is the first drug ever to target gangliosides on a structurally based drug design strategy [39,40]. Both its small size (Mw < 1400) and its amino acid sequence confer a very high solubility in water (>200 mg/mL–146 mM) (Table 1). Its cationic properties allow an efficient and rapid association with cell membranes, a property shared by cell-penetrating peptides (CPP) [101,102] that cross the blood–brain barrier [85]. 

Previously published toxicology data indicated that AmyP53 is very well tolerated in rats, as no undesirable effect could be detected at doses up to 5 mg/kg body weight (intranasal administration) or 80 mg/kg (intravenous administration) [75]. In particular, no inflammatory reaction could be observed in the nostrils and the tails of AmyP53-treated animals [75]. Herein, we provide the results of a complementary toxicology study conducted in four groups of eight rats (four males, 4 = four females) (Table 3), which further demonstrates the safety of AmyP53 intranasal administration [75]. Moreover, neural cells engineered to permanently secrete AmyP53 (AmyP53^+^ cells) were as healthy as control cells (AmyP53^−^ cells), although the culture supernatants of AmyP53^+^ cells contained high amounts of the biologically active AmyP53 peptide [75]. These data further demonstrated the safety of the long-term chronic exposure of neural cells to AmyP53. To complete the preclinical characterization of AmyP53, we provide herein stability and pharmacokinetics data, as well as the first demonstration that AmyP53 can reach the brain after either intranasal or intravenous administration. 

The chemical stability of AmyP53 in water solution was studied at three temperatures (4 °C, 20 °C, and 45 °C) for a total period of 18 months. We found that AmyP53 remained remarkably stable under all these conditions (Figure 2). These data demonstrated that the peptide bonds of AmyP53 behaved as expected for synthetic peptides, which, among other advantages, are generally stable at room temperature [103,104]. Nevertheless, a potential drawback is that chemical stability may not always correlate with biological activity, since the molecule could, for instance, self-aggregate during its storage [105]. Thus, we checked that the stored solutions contained not only chemically intact but also biologically active AmyP53. To this end, we carefully evaluated the ganglioside-binding capacity of stored peptide solutions, which indeed was the case at all temperatures tested during the whole 18-month incubation period (Figure 3). 

Then we studied the apparent stability of AmyP53 in whole blood and in serum (Figure 4). In agreement with previous data obtained with synthetic peptides, AmyP53 was more stable in whole blood than in serum [106], with half-life times of 17 min and 5 min, respectively. This difference may reflect a protective effect of red blood cells that could probably adsorb AmyP53 on their own plasma membrane gangliosides [107]. One could also consider the possibility that AmyP53 could bind to serum albumin [108], which could decrease the yield of extraction by methanol. Future studies will help clarify this issue. In any case, it should be noted that AmyP53 does not display any identified cleavage site for blood proteases. Finally, the half-life times determined for AmyP53 in whole blood and serum were consistent with comparable peptides [109,110,111,112,113].

**Figure 8:**
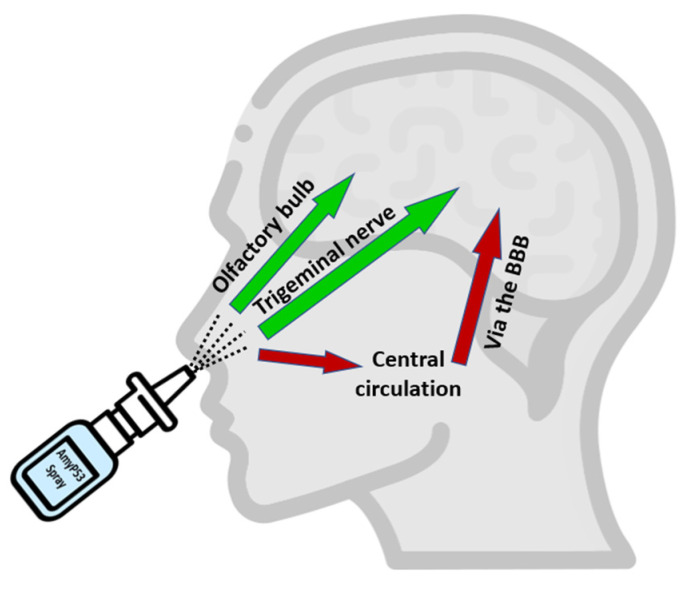
Pathways of AmyP53 intranasal transport. Following intranasal administration, AmyP53 can reach the brain by direct and indirect pathways. The direct pathway may occur via the trigeminal nerve or the olfactory bulbs. The indirect pathway may involve successively blood circulation and the blood–brain barrier (BBB).

Following a single intranasal administration of AmyP53, the peptide was rapidly detected in the brain (Figure 5B), suggesting an efficient transport via the direct nose-to-brain pathway [94] (Figure 8). In comparison, AmyP53 could also reach the brain tissue after intravenous administration but after a lag of 15 min (Figure 5B). The presence of AmyP53 in the brain after intravenous administration strongly suggests that the peptide is able to cross the blood–brain barrier. To check this possibility, we used in vitro reconstituted models of the blood–brain barrier [77,114] and compared the transendothelial transport of AmyP53 with two cargo peptides, synB3 and synB5 (Figure 6). Interestingly, all three peptides shared a common amino acid signature consisting of a central tyrosine residue and lateral basic amino acids. We confirmed that AmyP53 efficiently crosses the blood–brain barrier, at least as efficiently as syn B3 and even more than syn B5. In fact, we could establish a relationship between the size of the peptides and their kinetics of transport through the blood–brain barrier, the shortest being the best. Moreover, AmyP53 retained its therapeutic activity after the blood–brain barrier transport, as tested in the amyloid pore assay (Figure 7). Overall, these data gave a mechanistic explanation for the detection of AmyP53 in the brain after intravenous administration. On the other hand, AmyP53 was consistently detected in circulating blood after intranasal administration, which may also suggest some transport through the indirect pathway [94] (Figure 8).

The pharmacokinetics of AmyP53 in the brain suggest a rapid decline of the peptide after the initial peak at 5 min, but the peptide was still detected after 2 h. The minimal fully active concentration of AmyP53 has been determined in the amyloid-pore-forming assay at 50 nM in presence of a large excess of amyloid proteins [75]. Thus, the amount of AmyP53 detected in the brain after a single intranasal administration (Table 2) is significantly higher than the dose required for a therapeutic effect. On the basis of these data, we definitely selected the intranasal administration for delivering AmyP53 to the brain with a dedicated nasal-spray device. Indeed, AmyP53 is highly soluble in water and stable over storage, and it efficiently and rapidly reaches the brain tissue through the nose-to-brain pathway. This mode of delivery is obviously particularly comfortable for patients and healthcare workers, but it also bypasses the potential issues associated with the impairments of the blood–brain barrier in patients with neurodegenerative diseases [115]. Among the therapeutic peptides delivered to the brain via the nose-to-brain pathway (e.g., vasopressin [116], VIP [117], PACAP [118], TGF-β [119], NGF [120], EPO [121], or β-IFN [122]), a common parameter seems to be the cationic charge since all of these peptides have an isoelectric point (pH_i_) higher than eight (Table 4). The number of amino acids is not discriminant since the list includes both short and large peptides (from 9 to 166 residues). With a pH_i_ of 8.5 and a length of 12 amino acids, AmyP53 is ideally placed in this list.

Finally, according to the therapeutic peptide database THPdb [123], which lists all FDA-approved therapeutic peptides and proteins, there are currently 11% of peptides with 1–30 amino acids and 2% for neurological diseases. Interestingly, a large ratio of therapeutic peptides (46%) are directed against receptors, but none against gangliosides, which remains a distinctive innovation of AmyP53. 

## 4. Materials and Methods

### 4.1. Materials

AmyP53 (purity >98%), free of endotoxin and residual solvents, was synthesized by Proteogenix (Schiltigheim, France). Stock solutions (up to 200 mg/mL) were prepared in HPLC-grade water and stored at −20 °C before use. Cell-penetrating peptides synB3 and synB5 (17-mer) (purity >95%) were purchased from SchaferN (Copenhagen, Denmark). Ganglioside GM1 was purchased from Matreya (State College, PA, USA).

### 4.2. Cell Penetrating Prediction Method

The amino acid sequence of AmyP53 was submitted to the C2Pred server, a sequence-based tool for identifying cell-penetrating peptides (CPP) [86]. 

### 4.3. Microtensiometry (Ganglioside Binding Assay)

The interaction of AmyP53 with ganglioside monolayers was analyzed at the air–water interface with a dedicated tensiometer (Kibron µTrough, Helsinki, Finland) as described previously [74,124].

### 4.4. Animals

Male and female Sprague Dawley rats, 6 weeks old at receipt, weighing around 300 g at the beginning of the experiments were used for pharmacokinetics studies. The animals that were included in these experiments were naïve to the previous administration of drugs. The acclimatization of rats lasted at least 5 days. Animals had free access to food (RM1, SDS Dietex) and drinking water ad libitum. Animal studies were conducted by Syncrosome (Marseille, France) for pharmacokinetics studies and by EtapLab (Vandoeuvre-les-Nancy, France) for toxicology studies. The protocol used by Syncrosome was approved by an Animal Ethical Committee (French National Committee N°71) and by the Higher Education and Research Ministry (#2015070314429284_v2). The Toxicology Department of EtapLab has obtained the ISO 9001:v2008 certification for “Consulting, advising expertise in toxicology, studies in toxicology performed or conducted by the Toxicology Department”. The experiments performed by EtapLab have been approved by the CELMEA ethics committee (AMYPORE/P4-T-0919/AmyP53/DRF-IN/v1 and AMYPORE/P3-T-0719/AmyP53/IV-IN/v1) and the study has been carried out in compliance with the ARRIVE guidelines. Every effort was done to minimize animal suffering and to reduce the number of animals used in the experiments in compliance with the ARRIVE guidelines.

### 4.5. Toxicology Studies

Male and female Wistar rats (4 animals per group) were treated with the indicated dose of AmyP53, and toxicology studies were conducted as previously described [75]. Statistical analysis was performed with the Kruskal–Wallis test.

### 4.6. Pharmacokinetics Studies

One compound formulation at one dose for each route of administration was tested during the present study. The data were treated with the PKsolver add-in of Excel [125]. 

### 4.7. Intravenous (IV) Administration

Anesthetized (ketamine/medetomidine or isoflurane 4% for induction and then 2.5%) rats were treated intravenously once. The final volume of administration was 0.5 mL/kg (100 mg/kg body weight). As soon as the administration was performed (t_0_), a timer was started.

### 4.8. Intranasal (IN) Administration for Nose to Brain Delivery

A catheter was introduced into each nostril of the rat with a depth of 7 mm [126]. Anesthetized (ketamine/medetomidine or isoflurane 4% for induction and then 2.5%) rats were treated once. The volume of administration was 10 μL/nostril (20 μL per rat, 12 mg/kg body weight). As soon as the administration was performed (t_0_), a timer was started.

### 4.9. Blood Sampling and Plasma Preparation

Blood samples of 2 mL were collected through direct intracardiac puncture and separated on two K3-EDTA Greiner Bio-One tubes of 1 mL (ref. 454034A, Dominique Dutscher, Bernolsheim, France). A 100× solution of protease inhibitors (ref. 1862209, ThermoScientific, Waltham, MA, USA) was added immediately after sampling (10 μL/tube). The first tube of the blood sample was then separated in 2 samples with different procedures (with and without sonication) and the other one was used for plasma preparation. 

### 4.10. Brain Collection

Brains were collected immediately after blood collection, split in 2 hemispheres, and then transferred into Eppendorf tubes. Brain samples were placed into crushed dry ice for several minutes and frozen at −80 °C. When indicated, the rats were transcardially perfused with a solution of phosphate-buffered saline (PBS) for 10 min with a flow rate of 40 mL/min to remove blood in the brain tissue. A subcutaneous administration of buprenorphine (0.03 mg/kg) was performed at least 30 min prior to transcardial perfusion. 

### 4.11. AmyP53 Extraction

For blood and serum stability and for pharmacokinetics studies, blood and plasma samples (200 µL) were incubated with 3 volumes of methanol for 30 min at 4 °C and then centrifuged at 4 °C for 5 min (16,000× *g*). Supernatants were stored at −20 °C before analysis. Brain homogenates were prepared at 4 °C in PBS containing 1 mM EDTA and protease inhibitors (ref. 1862209, ThermoScientific, Waltham, MA, USA) in a glass Potter homogenizer. Homogenate samples (600 µL) were incubated with 3 volumes of methanol for 30 min at 4 °C and centrifuged at 4 °C for 5 min (16,000× *g*). Supernatants were evaporated under nitrogen flux and then diluted in 100 µL of 0.1% formic acid in water. 

### 4.12. AmyP53 LC–MS Measurements

The concentrations of intact AmyP53 in biological samples were determined by liquid chromatography–mass spectrometry (Acquity iClass-Vion LC-MS; Waters, Guyancourt, France). A total of 1–10 μL of each extract was injected into a C18 reverse phase column (BEH 2.1 × 50 mm, 1.7 μm; elution gradient H2O/CH3CN 0.1% formic acid). The injection needle was rinsed with 20% isopropanol (to avoid memory effects). Positive-mode electrospray ionization was performed at 2.5 kV/40 V, and AmyP53 [M+3H]3+ major ions were selectively monitored by a Tof-MRM method using 3 fragmentation transitions (456.58 → 527.32; 456.58 → 579.30; 456.58 → 841.43). CID fragmentations were operated at 20 eV for the three transitions. Data processing was performed with the UNIFI software (Waters, Milford, MA, USA). Quadratic external calibration was performed with AmyP53 standards (in 0.1% formic acid water) for sample concentration estimation.

### 4.13. In Vitro AmyP53 Stability Studies

AmyP53 samples (1 mM in water) were stored at 4 °C, 20 °C, and 45 °C in sealed Eppendorf tubes and analyzed at different time points for chemical integrity (LC-MS method) and biological activity (binding to ganglioside GM1). For calibration, different dilutions of the standard solution were injected three times, and the peak area was measured for all three. The % RSD for the area of three replicate injections was found to be within the specified limits [127]. The percentage of recovery was >98%, in full agreement with the certificate analysis of AmyP53 batch # #P190628-LL735654. LC–MS linearity was observed over the 5–100 pmol injected range (R^2^ > 0.99). The minimal amount detected was 5 pmol.

### 4.14. AmyP53 Stability in Blood and Serum

AmyP53 in water solution (300 µM) was mixed with rat blood (obtained from Syncrosome, Marseille, France), or commercial human serum (Sigma-Aldrich, St. Louis, MO, USA). AmyP53 concentrations were then determined by LC–MS at the indicated times after extraction, as detailed above.

### 4.15. Blood–Brain Barrier Studies

An in vitro model of the blood–brain barrier was reconstituted with bEnd.3 murine endothelial cells obtained from the American Type Culture Collection (ATCC, Manassas, VA, USA) ref. bEnd.3 [BEND3] (ATCC CRL-2299). These cells were cultured in chambers with two compartments separated by a filter with a porosity of 0.4 µm (Corning-Transwell, Dominique Dutscher, Bernolsheim, France). The cells were seeded at a density of 50,000 cells per individual filter and cultured in a growth medium containing 10% fetal calf serum. After an initial spreading step, the bEnd.3 cells colonized the entire surface available on the filter, thus reconstituting a functional endothelium. At this stage, the cells established tight junctions preventing the passage of molecules and ions. In order to improve the differentiation of bEnd.3 cells, we also set up two coculture systems: [bEnd.3 + astrocytes (CTX cells)] and [bEnd.3 + glioma (C6 cells)]. In these cases, the glial cells were seeded in the basal compartment (non-contact model) [114] and co-cultured for 6 days before using bEnd.3 cell monolayers for transport experiments. The impermeability of each of these cellular barriers was assessed by transendothelial resistance measurements with a Voltmeter-Ohmmeter Millicell-ERS (Merck-Millipore, Burlington, MA, USA). The tested peptides (or bovine serum albumin) were injected in the lower compartment and their concentration in the upper compartment was analyzed as a function of time by spectrophotometry. All experiments were performed 6 times.

### 4.16. Calcium Flux Measurements (Amyloid Pore Assay)

SH-SY5Y cells were used as a model for testing the amyloid-pore-blocking properties of AmyP53 [60,73] recovered from the acceptor compartment of reconstituted blood–brain-barrier systems. The cells were loaded with 5 μM Fluo-4AM for 30 min in the dark, washed three times with HBSS, and incubated for 30 min at 37 °C. Calcium fluxes by Aβ1-42 were measured as described previously [75] in the absence or presence of AmyP53^−^ or AmyP53^+^ culture supernatants (competition experiment). Signals were expressed as fluorescence after treatment (F_t_) divided by the fluorescence before treatment (F_t0_) multiplied by 100. All experiments were performed at 30 °C. All experiments were performed 6 times. Statistical analysis was performed with the Kruskal–Wallis test. 

## 5. Conclusions

Overall, we showed that AmyP53 is a cationic peptide, is highly soluble in water, and is remarkably stable upon storage in aqueous solution. It can reach the brain after intranasal administration via two distinct routes: (i) the direct pathway (through the olfactory bulbs and/or the trigeminal nerve) and (ii) the indirect pathway (via the systemic circulation and transport to the brain by transport through the blood–brain barrier) [94,128]. This therapeutic peptide can thus be formulated in a nasal spray for delivery via the nose-to-brain route, a noninvasive solution especially well adapted to the treatment of brain diseases [129,130,131,132,133,134]. 

## Figures and Tables

**Table 1:** Physicochemical properties of AmyP53.

Amino Acid Sequence	KEGVLYVGHHTK
Molecular weight	1367.57
Powder color	white
Isoelectric point (pHi)	8.5
Solubility in water	>146 mM (200 mg.mL^−1^)
Spectroscopic signature	A230/A275 = 6

**Table 2:** Pharmacokinetics parameters following the intravenous and nasal administration of AmyP53 in rats.

Parameter	Route	Blood	Brain
Cmax (ng/mL)	Intranasal (IN) Intravenous (IV)	347 254	1766 1095
Tmax (min)	IN IV	10 5	5 15
AUC 0→120 (ng.mL/min)	IN IV	15,240 12,342	62,794 67,140
Ratio of AUC_IN_/AUC_iv_ (%)		123	94
T1/2 (min)	IN IV	132 114	61 547
AmyP53 reaching the brain (%)	IN IV	- -	1.57 0.20

**Table 3:** Safety data relative to the intranasal administration of AmyP53 in rats.

Group and Dose (mg/kg)	Number of Animals	Inflammation at Nostrils	Body Weight (Day 7)	Behavior (Days 1–7)	Brain Histology ^2^
Vehicle 0	Male 4 Female 4	No No	246.99 ± 3.41 192.46 ± 5.44	NTR ^1^ NTR	NTR NTR
G1 0.2	Male 4 Female 4	No No	248.94 ± 8.51 189.46 ± 15.15	NTR NTR	NTR NTR
G2 1.0	Male 4 Female 4	No No	252.26 ± 5.77 191.03 ± 3.13	NTR NTR	NTR NTR
G3 5.0	Male 4 Female 4	No No	250.90 ± 5.68 185.70 ± 5.77	NTR NTR	NTR NTR

Male and female rats of the 4 experimental groups injected intranasally with doses of aqueous solutions of AmyP53 of 0.2, 1.0, and 5.0 mg/kg body weight every two days during 7 days. ^1^ NTR, nothing to report. ^2^ Brain histology. Data are expressed as mean ± SEM (n = 4). The differences in body weight between animals treated with vehicle or with AmyP53 were statistically not significant (*p* > 0.05, Kruskal–Wallis test).

**Table 4:** Therapeutic peptides delivered to the brain via the intranasal route.

Peptide	AmyP53	Vasopressin	VIP	PACAP	TGF-β	NGF	EPO	β-IFN
Length (aa)	12	9	28	38	112	120	165	166
pHi	8.5	8.0	9.8	10.4	8.6	9.0	8.7	8.9

## References

[1] Calabrò M., Rinaldi C., Santoro G., Crisafulli C. (2021). The biological pathways of Alzheimer disease: A review. AIMS Neurosci..

[2] Armstrong M.J., Okun M.S. (2020). Diagnosis and Treatment of Parkinson Disease: A review. JAMA.

[3] Collaborators G.N. (2019). Global, regional, and national burden of neurological disorders, 1990–2016: A systematic analysis for the Global Burden of Disease Study 2016. Lancet Neurol..

[4] Snowdon D.A. (1997). Aging and Alzheimer’s disease: Lessons from the Nun Study. Gerontologist.

[5] Tomlinson B.E., Blessed G., Roth M. (1968). Observations on the brains of non-demented old people. J. Neurol. Sci..

[6] Goure W.F., A Krafft G., Jerecic J., Hefti F. (2014). Targeting the proper amyloid-beta neuronal toxins: A path forward for Alzheimer’s disease immunotherapeutics. Alzheimer’s Res. Ther..

[7] Karran E., Hardy J. (2014). A critique of the drug discovery and phase 3 clinical programs targeting the amyloid hypothesis for Alzheimer disease. Ann. Neurol..

[8] Makin S. (2018). The amyloid hypothesis on trial. Nature.

[9] Mintun M.A., Lo A.C., Duggan Evans C., Wessels A.M., Ardayfio P.A., Andersen S.W., Shcherbinin S., Sparks J., Sims J.R., Brys M. (2021). Donanemab in Early Alzheimer’s Disease. N. Engl. J. Med..

[10] Tolar M., Hey J., Power A., Abushakra S. (2021). Neurotoxic Soluble Amyloid Oligomers Drive Alzheimer’s Pathogenesis and Represent a Clinically Validated Target for Slowing Disease Progression. Int. J. Mol. Sci..

[11] Knopman D.S., Jones D.T., Greicius M.D. (2021). Failure to demonstrate efficacy of aducanumab: An analysis of the EMERGE and ENGAGE trials as reported by Biogen, December 2019. Alzheimer’s Dement. J. Alzheimer’s Assoc..

[12] Tomiyama T., Nagata T., Shimada H., Teraoka R., Fukushima A., Kanemitsu H., Takuma H., Kuwano R., Imagawa M., Ataka S. (2008). A new amyloid beta variant favoring oligomerization in Alzheimer’s-type dementia. Ann. Neurol..

[13] Shimada H., Ataka S., Tomiyama T., Takechi H., Mori H., Miki T. (2011). Clinical course of patients with familial early-onset Alzheimer’s disease potentially lacking senile plaques bearing the E693Δ mutation in amyloid precursor protein. Dement. Geriatr. Cogn. Disord..

[14] Crystal H., Dickson D., Fuld P., Masur D., Scott R., Mehler M., Masdeu J., Kawas C., Aronson M., Wolfson L. (1988). Clinico-pathologic studies in dementia: Nondemented subjects with pathologically confirmed Alzheimer’s disease. Neurology.

[15] Dickson D.W., Crystal H.A., Mattiace L.A., Masur D.M., Blau A.D., Davies P., Yen S.H., Aronson M.K. (1992). Identification of normal and pathological aging in prospectively studied nondemented elderly humans. Neurobiol. Aging.

[16] Delaère P., He Y., Fayet G., Duyckaerts C., Hauw J.J. (1993). Beta A4 deposits are constant in the brain of the oldest old: An immunocytochemical study of 20 French centenarians. Neurobiol. Aging.

[17] Haass C., Selkoe D.J. (2007). Soluble protein oligomers in neurodegeneration: Lessons from the Alzheimer’s amyloid beta-peptide. Nat. Rev. Mol. Cell Biol..

[18] Kayed R., Pensalfini A., Margol L., Sokolov Y., Sarsoza F., Head E., Hall J., Glabe C. (2009). Annular protofibrils are a structurally and functionally distinct type of amyloid oligomer. J. Biol. Chem..

[19] Esparza T.J., Zhao H., Cirrito J.R., Cairns N.J., Bateman R.J., Holtzman D.M., Brody D.L. (2013). Amyloid-β oligomerization in Alzheimer dementia versus high-pathology controls. Ann. Neurol..

[20] Bode D.C., Baker M.D., Viles J.H. (2017). Ion Channel Formation by Amyloid-β42 Oligomers but Not Amyloid-β40 in Cellular Membranes. J. Biol. Chem..

[21] Cline E.N., Bicca M.A., Viola K.L., Klein W.L. (2018). The Amyloid-β Oligomer Hypothesis: Beginning of the Third Decade. J. Alzheimer’s Dis..

[22] Huang Y.R., Liu R.T. (2020). The Toxicity and Polymorphism of β-Amyloid Oligomers. Int. J. Mol. Sci..

[23] Gonzalez-Garcia M., Fusco G., de Simone A. (2021). Membrane Interactions and Toxicity by Misfolded Protein Oligomers. Front. Cell Dev. Biol..

[24] Gaig C., Martí M.J., Ezquerra M., Rey M.J., Cardozo A., Tolosa E. (2007). G2019S LRRK2 mutation causing Parkinson’s disease without Lewy bodies. J. Neurol. Neurosurg. Psychiatry.

[25] Colosimo C., Hughes A.J., Kilford L., Lees A.J. (2003). Lewy body cortical involvement may not always predict dementia in Parkinson’s disease. J. Neurol. Neurosurg. Psychiatry.

[26] Parkkinen L., Kauppinen T., Pirttilä T., Autere J.M., Alafuzoff I. (2005). Alpha-synuclein pathology does not predict extrapyramidal symptoms or dementia. Ann. Neurol..

[27] Winner B., Jappelli R., Maji S.K., Desplats P.A., Boyer L., Aigner S., Hetzer C., Loher T., Vilar M., Campioni S. (2011). In vivo demonstration that alpha-synuclein oligomers are toxic. Proc. Natl. Acad. Sci. USA.

[28] Schmidt F., Levin J., Kamp F., Kretzschmar H., Giese A., Bötzel K. (2012). Single-Channel Electrophysiology Reveals a Distinct and Uniform Pore Complex Formed by α-Synuclein Oligomers in Lipid Membranes. PLoS ONE.

[29] Angelova P.R., Ludtmann M.H., Horrocks M.H., Negoda A., Cremades N., Klenerman D., Dobson C.M., Wood N.W., Pavlov E.V., Gandhi S. (2016). Ca^2+^ is a key factor in α-synuclein-induced neurotoxicity. J. Cell Sci..

[30] Ingelsson M. (2016). Alpha-Synuclein Oligomers-Neurotoxic Molecules in Parkinson’s Disease and Other Lewy Body Disorders. Front. Neurosci..

[31] Rockenstein E., Nuber S., Overk C.R., Ubhi K., Mante M., Patrick C., Adame A., Trejo-Morales M., Gerez J., Picotti P. (2014). Accumulation of oligomer-prone α-synuclein exacerbates synaptic and neuronal degeneration in vivo. Brain J. Neurol..

[32] Bengoa-Vergniory N., Roberts R.F., Wade-Martins R., Alegre-Abarrategui J. (2017). Alpha-synuclein oligomers: A new hope. Acta Neuropathol..

[33] Cascella R., Bigi A., Cremades N., Cecchi C. (2022). Effects of oligomer toxicity, fibril toxicity and fibril spreading in synucleinopathies. Cell. Mol. Life Sci..

[34] Gutierrez B.A., Limon A. (2022). Synaptic Disruption by Soluble Oligomers in Patients with Alzheimer’s and Parkinson’s Disease. Biomedicines.

[35] Kayed R., Head E., Thompson J.L., McIntire T.M., Milton S.C., Cotman C.W., Glabe C.G. (2003). Common structure of soluble amyloid oligomers implies common mechanism of pathogenesis. Science.

[36] Arispe N., Rojas E., Pollard H.B. (1993). Alzheimer disease amyloid beta protein forms calcium channels in bilayer membranes: Blockade by tromethamine and aluminum. Proc. Natl. Acad. Sci. USA.

[37] Jang H., Connelly L., Arce F.T., Ramachandran S., Lal R., Kagan B.L., Nussinov R. (2013). Alzheimer’s disease: Which type of amyloid-preventing drug agents to employ?. Phys. Chem. Chem. Phys..

[38] Jang H., Arce F.T., Ramachandran S., Capone R., Azimova R., Kagan B.L., Nussinov R., Lal R. (2010). Truncated beta-amyloid peptide channels provide an alternative mechanism for Alzheimer’s Disease and Down syndrome. Proc. Natl. Acad. Sci. USA.

[39] Fantini J., Chahinian H., Yahi N. (2020). Progress toward Alzheimer’s disease treatment: Leveraging the Achilles’ heel of Aβ oligomers?. Protein Sci. Publ. Protein Soc..

[40] Yahi N., Di Scala C., Chahinian H., Fantini J. (2022). Innovative treatment targeting gangliosides aimed at blocking the formation of neurotoxic α-synuclein oligomers in Parkinson’s disease. Glycoconj. J..

[41] Lukiw W.J. (2013). Alzheimer’s disease (AD) as a disorder of the plasma membrane. Front. Physiol..

[42] Fabiani C., Antollini S.S. (2019). Alzheimer’s Disease as a Membrane Disorder: Spatial Cross-Talk Among Beta-Amyloid Peptides, Nicotinic Acetylcholine Receptors and Lipid Rafts. Front. Cell. Neurosci..

[43] Schnaar R.L. (2016). Gangliosides of the Vertebrate Nervous System. J. Mol. Biol..

[44] Fantini J., Yahi N. (2015). Brain Lipids in Synaptic Function and Neurological Disease: Clues to Innovative Therapeutic Strategies for Brain Disorders.

[45] Fantini J., Garmy N., Mahfoud R., Yahi N. (2002). Lipid rafts: Structure, function and role in HIV, Alzheimer’s and prion diseases. Expert Rev. Mol. Med..

[46] Kawarabayashi T., Nakamura T., Sato K., Seino Y., Ichii S., Nakahata N., Takatama M., Westaway D., George-Hyslop P.S., Shoji M. (2022). Lipid Rafts Act as a Common Platform for Amyloid-β Oligomer-Induced Alzheimer’s Disease Pathology. J. Alzheimer’s Dis..

[47] Hong S., Ostaszewski B.L., Yang T., O’Malley T.T., Jin M., Yanagisawa K., Li S., Bartels T., Selkoe D.J. (2014). Soluble Aβ Oligomers Are Rapidly Sequestered from Brain ISF In Vivo and Bind GM1 Ganglioside on Cellular Membranes. Neuron.

[48] Fernández-Pérez E.J., Sepúlveda F.J., Peoples R., Aguayo L.G. (2017). Role of membrane GM1 on early neuronal membrane actions of Aβ during onset of Alzheimer’s disease. Biochim. et Biophys. Acta Mol. Basis Dis..

[49] Ledeen R.W., Wu G. (2018). Gangliosides, α-Synuclein, and Parkinson’s Disease. Prog. Mol. Biol. Transl. Sci..

[50] Sipione S., Monyror J., Galleguillos D., Steinberg N., Kadam V. (2020). Gangliosides in the Brain: Physiology, Pathophysiology and Therapeutic Applications. Front. Neurosci..

[51] Venko K., Novič M., Stoka V., Žerovnik E. (2021). Prediction of Transmembrane Regions, Cholesterol, and Ganglioside Binding Sites in Amyloid-Forming Proteins Indicate Potential for Amyloid Pore Formation. Front. Mol. Neurosci..

[52] Matsuzaki K. (2020). Aβ-ganglioside interactions in the pathogenesis of Alzheimer’s disease. Biochim. Biophys. Acta Biomembr..

[53] Yanagisawa K. (2015). GM1 ganglioside and Alzheimer’s disease. Glycoconj. J..

[54] Fantini J., Yahi N., Garmy N. (2013). Cholesterol accelerates the binding of Alzheimer’s β-amyloid peptide to ganglioside GM1 through a universal hydrogen-bond-dependent sterol tuning of glycolipid conformation. Front. Physiol..

[55] Kaur U., Lee J.C. (2020). Unroofing site-specific α-synuclein-lipid interactions at the plasma membrane. Proc. Natl. Acad. Sci. USA.

[56] Perissinotto F., Rondelli V., Parisse P., Tormena N., Zunino A., Almásy L., Merkel D.G., Bottyán L., Sajti S., Casalis L. (2019). GM1 Ganglioside role in the interaction of Alpha-synuclein with lipid membranes: Morphology and structure. Biophys. Chem..

[57] Fantini J., Yahi N. (2011). Molecular Basis for the Glycosphingolipid-Binding Specificity of α-Synuclein: Key Role of Tyrosine 39 in Membrane Insertion. J. Mol. Biol..

[58] Fantini J., Yahi N. (2013). The driving force of alpha-synuclein insertion and amyloid channel formation in the plasma membrane of neural cells: Key role of ganglioside- and cholesterol-binding domains. Adv. Exp. Med. Biol..

[59] Lashuel H.A., Hartley D., Petre B.M., Walz T., Lansbury P.T. (2002). Neurodegenerative disease: Amyloid pores from pathogenic mutations. Nature.

[60] Di Scala C., Yahi N., Boutemeur S., Flores A., Rodriguez L., Chahinian H., Fantini J. (2016). Common molecular mechanism of amyloid pore formation by Alzheimer’s β-amyloid peptide and α-synuclein. Sci. Rep..

[61] De Felice F.G., Wu D., Lambert M.P., Fernandez S.J., Velasco P.T., Lacor P.N., Bigio E.H., Jerecic J., Acton P.J., Shughrue P.J. (2008). Alzheimer’s disease-type neuronal tau hyperphosphorylation induced by A beta oligomers. Neurobiol. Aging.

[62] Zempel H., Thies E., Mandelkow E., Mandelkow E.M. (2010). Abeta oligomers cause localized Ca(2+) elevation, missorting of endogenous Tau into dendrites, Tau phosphorylation, and destruction of microtubules and spines. J. Neurosci. Off. J. Soc. Neurosci..

[63] Rudenko L.K., Wallrabe H., Periasamy A., Siller K.H., Svindrych Z., Seward M.E., Best M.N., Bloom G.S. (2019). Intraneuronal Tau Misfolding Induced by Extracellular Amyloid-β Oligomers. J. Alzheimer’s Dis..

[64] Tabner B.J., El-Agnaf O.M., Turnbull S., German M.J., Paleologou K.E., Hayashi Y., Cooper L.J., Fullwood N.J., Allsop D. (2005). Hydrogen peroxide is generated during the very early stages of aggregation of the amyloid peptides implicated in Alzheimer disease and familial British dementia. J. Biol. Chem..

[65] Deas E., Cremades N., Angelova P.R., Ludtmann M.H., Yao Z., Chen S., Horrocks M.H., Banushi B., Little D., Devine M.J. (2016). Alpha-Synuclein Oligomers Interact with Metal Ions to Induce Oxidative Stress and Neuronal Death in Parkinson’s Disease. Antioxid. Redox Signal..

[66] Lacor P.N., Buniel M.C., Furlow P.W., Clemente A.S., Velasco P.T., Wood M., Viola K.L., Klein W.L. (2007). Abeta oligomer-induced aberrations in synapse composition, shape, and density provide a molecular basis for loss of connectivity in Alzheimer’s disease. J. Neurosci. Off. J. Soc. Neurosci..

[67] Shankar G.M., Bloodgood B.L., Townsend M., Walsh D.M., Selkoe D.J., Sabatini B.L. (2007). Natural oligomers of the Alzheimer amyloid-beta protein induce reversible synapse loss by modulating an NMDA-type glutamate receptor-dependent signaling pathway. J. Neurosci. Off. J. Soc. Neurosci..

[68] Tu S., Okamoto S., Lipton S.A., Xu H. (2014). Oligomeric Aβ-induced synaptic dysfunction in Alzheimer’s disease. Mol. Neurodegener..

[69] Yang T., Li S., Xu H., Walsh D.M., Selkoe D.J. (2017). Large Soluble Oligomers of Amyloid β-Protein from Alzheimer Brain Are Far Less Neuroactive Than the Smaller Oligomers to Which They Dissociate. J. Neurosci. Off. J. Soc. Neurosci..

[70] Shankar G.M., Li S., Mehta T.H., Garcia-Munoz A., Shepardson N.E., Smith I., Brett F.M., Farrell M.A., Rowan M.J., Lemere C.A. (2008). Amyloid-beta protein dimers isolated directly from Alzheimer’s brains impair synaptic plasticity and memory. Nat. Med..

[71] Lambert M.P., Barlow A.K., Chromy B.A., Edwards C., Freed R., Liosatos M., Morgan T.E., Rozovsky I., Trommer B., Viola K.L. (1998). Diffusible, nonfibrillar ligands derived from Abeta1-42 are potent central nervous system neurotoxins. Proc. Natl. Acad. Sci. USA.

[72] Kim H.J., Chae S.C., Lee D.K., Chromy B., Lee S.C., Park Y.C., Klein W.L., Krafft G.A., Hong S.T. (2003). Selective neuronal degeneration induced by soluble oligomeric amyloid beta protein. FASEB J. Off. Publ. Fed. Am. Soc. Exp. Biol..

[73] Di Scala C., Yahi N., Flores A., Boutemeur S., Kourdougli N., Chahinian H., Fantini J. (2016). Broad neutralization of calcium-permeable amyloid pore channels with a chimeric Alzheimer/Parkinson peptide targeting brain gangliosides. Biochim. Biophys. Acta.

[74] Yahi N., Fantini J. (2014). Deciphering the glycolipid code of Alzheimer’s and Parkinson’s amyloid proteins allowed the creation of a universal ganglioside-binding peptide. PLoS ONE.

[75] El-Battari A., Rodriguez L., Chahinian H., Delézay O., Fantini J., Yahi N., Di Scala C. (2021). Gene Therapy Strategy for Alzheimer’s and Parkinson’s Diseases Aimed at Preventing the Formation of Neurotoxic Oligomers in SH-SY5Y Cells. Int. J. Mol. Sci..

[76] Fantini J., Yahi N., Chermann J.C. (1991). Human immunodeficiency virus can infect the apical and basolateral surfaces of human colonic epithelial cells. Proc. Natl. Acad. Sci. USA.

[77] Omidi Y., Campbell L., Barar J., Connell D., Akhtar S., Gumbleton M. (2003). Evaluation of the immortalised mouse brain capillary endothelial cell line, b.End3, as an in vitro blood-brain barrier model for drug uptake and transport studies. Brain Res..

[78] Fantini J., Verrier B., Marvaldi J., Mauchamp J. (1989). In vitro differentiated HT 29-D4 clonal cell line generates leakproof and electrically active monolayers when cultured in porous-bottom culture dishes. Biol. Cell.

[79] Fantini J., Rognoni J.B., Culouscou J.M., Pommier G., Marvaldi J., Tirard A. (1989). Induction of polarized apical expression and vectorial release of carcinoembryonic antigen (CEA) during the process of differentiation of HT29-D4 cells. J. Cell. Physiol..

[80] Gaillard P.J., de Boer A.G. (2000). Relationship between permeability status of the blood-brain barrier and in vitro permeability coefficient of a drug. Eur. J. Pharm. Sci. Off. J. Eur. Fed. Pharm. Sci..

[81] Maresca M., Mahfoud R., Garmy N., Kotler D.P., Fantini J., Clayton F. (2003). The virotoxin model of HIV-1 enteropathy: Involvement of GPR15/Bob and galactosylceramide in the cytopathic effects induced by HIV-1 gp120 in the HT-29-D4 intestinal cell line. J. Biomed. Sci..

[82] Brunet J.L., Maresca M., Fantini J., Belzunces L.P. (2004). Human intestinal absorption of imidacloprid with Caco-2 cells as enterocyte model. Toxicol. Appl. Pharmacol..

[83] Delsing L., Herland A., Falk A., Hicks R., Synnergren J., Zetterberg H. (2020). Models of the blood-brain barrier using iPSC-derived cells. Mol. Cell. Neurosci..

[84] Tewes B., Franke H., Hellwig S., Hoheisel D., Decker S., Griesche T., Tilling J. (1997). Preparation of endothelial cells in primary cultures obtained from the brains of 6-month-old pigs. Drug Transport Across the Blood-brain Barrier: In Vitro and In Vivo Techniques.

[85] Zou L., Ma J.-L., Wang T., Yang T.-B., Liu C.-B. (2013). Cell-Penetrating Peptide-Mediated Therapeutic Molecule Delivery into the Central Nervous System. Curr. Neuropharmacol..

[86] Tang H., Su Z.-D., Wei H.-H., Chen W., Lin H. (2016). Prediction of cell-penetrating peptides with feature selection techniques. Biochem. Biophys. Res. Commun..

[87] Lee A.C.-L., Harris J.L., Khanna K.K., Hong J.-H. (2019). A Comprehensive Review on Current Advances in Peptide Drug Development and Design. Int. J. Mol. Sci..

[88] Mitra A., Sarkar N. (2020). Sequence and structure-based peptides as potent amyloid inhibitors: A review. Arch. Biochem. Biophys..

[89] Morimoto B.H. (2018). Therapeutic peptides for CNS indications: Progress and challenges. Bioorganic Med. Chem..

[90] Apostolopoulos V., Bojarska J., Chai T.-T., Elnagdy S., Kaczmarek K., Matsoukas J., New R., Parang K., Lopez O.P., Parhiz H. (2021). A Global Review on Short Peptides: Frontiers and Perspectives. Molecules.

[91] Zane D., Feldman P.L., Sawyer T., Sobol Z., Hawes J. (2021). Development and Regulatory Challenges for Peptide Therapeutics. Int. J. Toxicol..

[92] Ribarič S. (2018). Peptides as Potential Therapeutics for Alzheimer’s Disease. Molecules.

[93] Sun L. (2013). Peptide-based drug development. Mod. Chem. Appl..

[94] Meredith M.E., Salameh T.S., Banks W.A. (2015). Intranasal Delivery of Proteins and Peptides in the Treatment of Neurodegenerative Diseases. AAPS J..

[95] Rat D., Schmitt U., Tippmann F., Dewachter I., Theunis C., Wieczerzak E., Postina R., van Leuven F., Fahrenholz F., Kojro E. (2011). Neuropeptide pituitary adenylate cyclase-activating polypeptide (PACAP) slows down Alzheimer’s disease-like pathology in amyloid precursor protein-transgenic mice. FASEB J. Off. Publ. Fed. Am. Soc. Exp. Biol..

[96] Dewji N.N., Azar M.R., Hanson L.R., Frey Ii W.H., Morimoto B.H., Johnson D. (2018). Pharmacokinetics in Rat of P8, a Peptide Drug Candidate for the Treatment of Alzheimer’s Disease: Stability and Delivery to the Brain. J. Alzheimer’s Dis. Rep..

[97] Wiciński M., Socha M., Malinowski B., Wódkiewicz E., Walczak M., Górski K., Słupski M., Pawlak-Osińska K. (2019). Liraglutide and its Neuroprotective Properties—Focus on Possible Biochemical Mechanisms in Alzheimer’s Disease and Cerebral Ischemic Events. Int. J. Mol. Sci..

[98] Qi L., Ke L., Liu X., Liao L., Ke S., Liu X., Wang Y., Lin X., Zhou Y., Wu L. (2016). Subcutaneous administration of liraglutide ameliorates learning and memory impairment by modulating tau hyperphosphorylation via the glycogen synthase kinase-3β pathway in an amyloid β protein induced alzheimer disease mouse model. Eur. J. Pharmacol..

[99] Liu W., Jalewa J., Sharma M., Li G., Li L., Hölscher C. (2015). Neuroprotective effects of lixisenatide and liraglutide in the 1-methyl-4-phenyl-1,2,3,6-tetrahydropyridine mouse model of Parkinson’s disease. Neuroscience.

[100] Badawi G.A., El Fattah M.A.A., Zaki H.F., El Sayed M.I. (2017). Sitagliptin and liraglutide reversed nigrostriatal degeneration of rodent brain in rotenone-induced Parkinson’s disease. Inflammopharmacology.

[101] Derakhshankhah H., Jafari S. (2018). Cell penetrating peptides: A concise review with emphasis on biomedical applications. Biomed. Pharmacother..

[102] Copolovici D.M., Langel K., Eriste E., Langel Ü. (2014). Cell-penetrating peptides: Design, synthesis, and applications. ACS Nano.

[103] Bozovičar K., Bratkovič T. (2020). Evolving a Peptide: Library Platforms and Diversification Strategies. Int. J. Mol. Sci..

[104] Lamers C. (2022). Overcoming the shortcomings of peptide-based therapeutics. Futur. Drug Discov..

[105] Li N.K., Xie Y., Yingling Y.G. (2021). Insights into Structure and Aggregation Behavior of Elastin-like Polypeptide Coacervates: All-Atom Molecular Dynamics Simulations. J. Phys. Chem. B.

[106] Böttger R., Hoffmann R., Knappe D. (2017). Differential stability of therapeutic peptides with different proteolytic cleavage sites in blood, plasma and serum. PLoS ONE.

[107] Conrard L., Stommen A., Cloos A.S., Steinkühler J., Dimova R., Pollet H., Tyteca D. (2018). Spatial Relationship and Functional Relevance of Three Lipid Domain Populations at the Erythrocyte Surface. Cell. Physiol. Biochem. Int. J. Exp. Cell. Physiol. Biochem. Pharmacol..

[108] Rózga M., Kłoniecki M., Jabłonowska A., Dadlez M., Bal W. (2007). The binding constant for amyloid Aβ40 peptide interaction with human serum albumin. Biochem. Biophys. Res. Commun..

[109] Das A., Urbanowski J., Weissbach H., Nestor J., Yanofsky C. (1983). In vitro synthesis of the tryptophan operon leader peptides of Escherichia coli, Serratia marcescens, and Salmonella typhimurium. Proc. Natl. Acad. Sci. USA.

[110] Oh J.E., Hong S.Y., Lee K.H. (1999). Design, synthesis and characterization of antimicrobial pseudopeptides corresponding to membrane-active peptide. J. Pept. Res. Off. J. Am. Pept. Soc..

[111] Adessi C., Frossard M.J., Boissard C., Fraga S., Bieler S., Ruckle T., Vilbois F., Robinson S.M., Mutter M., Banks W.A. (2003). Pharmacological profiles of peptide drug candidates for the treatment of Alzheimer’s disease. J. Biol. Chem..

[112] Kokko K.P., Hadden M.K., Orwig K.S., Mazella J., Dix T.A. (2003). In vitro analysis of stable, receptor-selective neurotensin[8–13] analogues. J. Med. Chem..

[113] Audsley N., Matthews J., Nachman R., Weaver R.J. (2007). Metabolism of cydiastatin 4 and analogues by enzymes associated with the midgut and haemolymph of Manduca sexta larvae. Gen. Comp. Endocrinol..

[114] Bicker J., Alves G., Fortuna A., Falcão A. (2014). Blood-brain barrier models and their relevance for a successful development of CNS drug delivery systems: A review. Eur. J. Pharm. Biopharm. Off. J. Arb. Fur Pharm. Verfahr. e.V.

[115] Sweeney M.D., Sagare A.P., Zlokovic B.V. (2018). Blood-brain barrier breakdown in Alzheimer disease and other neurodegenerative disorders. Nat. Rev. Neurol..

[116] Yang J., Lu L., Wang H.-C., Zhan H.-Q., Hai G.-F., Pan Y.-J., Lv Q.-Q., Wang D.-X., Wu Y.-Q., Li R.-R. (2012). Effect of intranasal arginine vasopressin on human headache. Peptides.

[117] Cui X., Cao D.-Y., Wang Z.-M., Zheng A.-P. (2013). Pharmacodynamics and toxicity of vasoactive intestinal peptide for intranasal administration. Pharm. Int. J. Pharm. Sci..

[118] Cherait A., Maucotel J., Lefranc B., Leprince J., Vaudry D. (2021). Intranasal Administration of PACAP Is an Efficient Delivery Route to Reduce Infarct Volume and Promote Functional Recovery After Transient and Permanent Middle Cerebral Artery Occlusion. Front. Endocrinol..

[119] Ma Y.-P., Ma M.-M., Ge S., Guo R.-B., Zhang H.-J., Frey W.H., Xu G.-L., Liu X.-F. (2007). Intranasally delivered TGF-β1 enters brain and regulates gene expressions of its receptors in rats. Brain Res. Bull..

[120] De Rosa R., Garcia A.A., Braschi C., Capsoni S., Maffei L., Berardi N., Cattaneo A. (2005). Intranasal administration of nerve growth factor (NGF) rescues recognition memory deficits in AD11 anti-NGF transgenic mice. Proc. Natl. Acad. Sci. USA.

[121] Merelli A., Caltana L., Lazarowski A., Brusco A. (2011). Experimental evidence of the potential use of erythropoietin by intranasal administration as a neuroprotective agent in cerebral hypoxia. Drug Metab. Drug Interact..

[122] Ross T.M., Martinez P.M., Renner J.C., Thorne R.G., Hanson L.R., Frey W.H. (2004). Intranasal administration of interferon beta bypasses the blood–brain barrier to target the central nervous system and cervical lymph nodes: A non-invasive treatment strategy for multiple sclerosis. J. Neuroimmunol..

[123] Usmani S.S., Bedi G., Samuel J.S., Singh S., Kalra S., Kumar P., Ahuja A.A., Sharma M., Gautam A., Raghava G.P.S. (2017). THPdb: Database of FDA-approved peptide and protein therapeutics. PLoS ONE.

[124] Di Scala C., Fantini J. (2017). Hybrid In Silico/In Vitro Approaches for the Identification of Functional Cholesterol-Binding Domains in Membrane Proteins. Methods Mol. Biol..

[125] Zhang Y., Huo M., Zhou J., Xie S. (2010). PKSolver: An add-in program for pharmacokinetic and pharmacodynamic data analysis in Microsoft Excel. Comput. Methods Programs Biomed..

[126] Stevens J., Suidgeest E., van der Graaf P.H., Danhof M., de Lange E.C.M. (2009). A New Minimal-Stress Freely-Moving Rat Model for Preclinical Studies on Intranasal Administration of CNS Drugs. Pharm. Res..

[127] Hubert P., Chiap P., Crommen J., Boulanger B., Chapuzet E., Mercier N., Bervoas-Martin S., Chevalier P., Grandjean D., Lagorce P. (1999). The SFSTP guide on the validation of chromatographic methods for drug bioanalysis: From the Washington Conference to the laboratory. Anal. Chim. Acta.

[128] Kozlovskaya L., Abou-Kaoud M., Stepensky D. (2014). Quantitative analysis of drug delivery to the brain via nasal route. J. Control. Release Off. J. Control. Release Soc..

[129] Umeda T., Tanaka A., Sakai A., Yamamoto A., Sakane T., Tomiyama T. (2018). Intranasal rifampicin for Alzheimer’s disease prevention. Alzheimer’s Dement..

[130] Gozes I., Giladi E., Pinhasov A., Bardea A., E Brenneman D. (2000). Activity-dependent neurotrophic factor: Intranasal administration of femtomolar-acting peptides improve performance in a water maze. J. Pharmacol. Exp. Ther..

[131] Stevens J., Ploeger B.A., van der Graaf P.H., Danhof M., de Lange E.C. (2011). Systemic and direct nose-to-brain transport pharmacokinetic model for remoxipride after intravenous and intranasal administration. Drug Metab. Dispos. Biol. Fate Chem..

[132] Agrawal M., Saraf S., Saraf S., Antimisiaris S.G., Chougule M.B., Shoyele S.A., Alexander A. (2018). Nose-to-brain drug delivery: An update on clinical challenges and progress towards approval of anti-Alzheimer drugs. J. Control. Release Off. J. Control. Release Soc..

[133] Erdő F., Bors L.A., Farkas D., Bajza Á., Gizurarson S. (2018). Evaluation of intranasal delivery route of drug administration for brain targeting. Brain Res. Bull..

[134] Trevino J.T., Quispe R.C., Khan F., Novak V. (2020). Non-Invasive Strategies for Nose-to-Brain Drug Delivery. J. Clin. Trials.

